# Supplementary material for: A meta-analysis of the association between male dimorphism and fitness outcomes in humans
Source: eLife. 2022 Feb 18;11:e65031. doi: 10.7554/eLife.65031 (PMC9106334; doi:10.7554/eLife.65031)
Supplement: Supplementary file 1. [file elife-65031-supp1.docx]

Supplementary File 1

*Effect size conversion formulas*

| Kendall’s tau | r = sin (.5 πτ) (Kendall, 1970) |
| --- | --- |
| Spearman's rho | not converted |
| t | rYλ = √(t^2^ / (t^2^ + df)) Online converter: <https://www.uccs.edu/lbecker/> |
| Odds ratio | Online converter: http://escal.site/ |
| Unstandardized regression coefficient (B) | β = (S.D. of predictor/S.D. of outcome) × B; r = .98β + .05λ (λ = 1 when β is nonnegative and 0 when β is negative) |
| Standardized regression coefficient (β) | r = .98β + .05λ (λ = 1 when β is nonnegative and 0 when β is negative; Peterson & Brown, 2005) |

**References**

M. G. Kendall, Rank correlation methods (4th ed.). (Charles Griffin & Co., 1970).

R. A. Peterson, S. P. Brown, On the use of beta coefficients in meta-analysis. *Adapt. Hum. Behav. Physiol.* 90(1), 175 (2005).
