# Supplementary material for: A meta-analysis of the association between male dimorphism and fitness outcomes in humans
Source: eLife. 2022 Feb 18;11:e65031. doi: 10.7554/eLife.65031 (PMC9106334; doi:10.7554/eLife.65031)
Supplement: Supplementary file 2. [file elife-65031-supp2.docx]

Supplementary File 2A

*General coding decisions*

| Sexual orientation | Coded as non-heterosexual sample if the sample was mixed but predominantly heterosexual. |
| --- | --- |
| Samples or subsamples comprising only fathers and/or married individuals | Coded as heterosexual unless otherwise specified, since they had reproduced/married heterosexually. |
| Student samples with a mean age ≤ 20 | Coded as non-fathers. |
| Sample contained ≥ 50% students | Coded as a student sample. |
| Sample contained both students and non-students but the proportion of students/non-students was not mentioned | Coded as a non-student sample. |
| Age | Considered an essential control for all outcome variables except age at first sexual intercourse/encounter and age at the birth of the first child, unless all participants were the same age, and for mating attitude measures. |
| Ethnicity | Coded as ‘white’ if ≥ 75% of sample was white. |
| Marriage system | Coded as polygynous if polygyny was permitted in population, even if rare. |
| Online samples | Coded as low fertility and monogamous. |
| Cut off point for high versus low fertility | 3.0 children/woman (in sample or population at the time of sampling). |
| Extreme outliers | Were included when possible, as outliers were expected. |
| Analyses of relevant relationships were included in paper, but authors had submitted results/raw data to us (e.g. results for men only, controlling for age etc.). | Coded as published results. |
| Preprints | Coded as non-published and non-peer-reviewed, unless the paper was later accepted for publication in which case it was updated as published and peer-reviewed. |
| Paper contained both zero-order correlations and multiple regression coefficients | We chose the regression coefficient if the multiple regression included relevant control variables (such as age), and the correlation coefficient if the multiple regression included irrelevant control variables. |
| Effect sizes given as Spearman’s rho | Were not converted; however, were coded as converted for moderation analyses, because it was not given as *r* and therefore considered to be an estimate. |
| Number of children in industrialized populations | Coded as children born (rather than surviving children) unless otherwise specified. (In naturally fertile populations, it is typically spelled out whether measures refer to number of children born vs number of surviving children.) |
| Dataset on age at first sexual intercourse contained virgins | Current age was used. |
| Testosterone studies where the authors only included samples that were clear | Coded as having controlled for blood contamination. |
| Muscularity measures | When other-rated, adiposity should be controlled for and was thus considered a necessary control; when own-rated, adiposity was not considered a necessary control, since people should be able to assess their own amount of muscle/adiposity. |
| Handgrip strength | Moderator ‘number of measurements’ was coded as number of measurements per hand, not in total. |

Supplementary File 2B

*Study-specific coding decisions*

| Authors | Decisions |
| --- | --- |
| Alvergne et al., 2009 | We assumed N=53 (married fathers only) as *p*-value does not add up if whole sample of married and non-married was analyzed together. It also makes sense to only analyze married men as they were the only ones who were able to reproduce. Not explicit in papers which variables were transformed to normality. |
| Apicella, 2014 | We excluded DVs >1 spouse in lifetime (considered redundant) and number of offspring born (effect size is the same for another variable but *p*-value differs - N is not stated, suggesting that either the effect size is not correct or N is considerably smaller). N for some analyses is not given, we assumed it was 51 as given for one of the analyses. They classified predictor as strength, but we re-coded it as a composite measure of muscle mass and handgrip strength as that is what it was (predictor was therefore not classified as either muscle mass or strength for moderation analyses). Some relationships reported in other papers as well: Smith, Olkhov, Puts & Apicella (2017) reported muscle mass/strength – reproductive success and offspring number; we kept results from this paper as it controlled for age, with the exclusion of offspring number for reason given above. |
| Apicella et al., 2007 | Some relationships reported in other papers as well: Smith, Olkhov, Puts & Apicella (2017) reported f0 - reproductive success and offspring number; we kept results from this paper as it controlled for age. |
| Arnocky et al., 2018 | Paper also included fWHR-lower - SOI-R and lower face/face height - SOI-R but effect sizes not reported separately for men and women so not included. |
| Atkinson et al., 2012 | Paper included both DVs number of living children and genetic vector; the latter calculated as 1*(number of living children) + ½*(number of living grandchildren). Considered redundant to include both, and to be consistent with other measures, we included number of living children. |
| Boothroyd et al., 2017 | Agta sample: photographs taken from front or ¾ degree angle were coded as not frontal photographs. |
| Charles & Alexander, 2011 | We excluded SOI (Clark, 2004) as it is redundant to SOI and SOI is the commonly used measure. Sample assumed to be non-fathers. |
| Falcon, 2016 | We used average 2D:4D rather than R2D:4D/L2D:4D due to bigger sample size and we could not rule out the possibility of overlapping samples. |
| Farrelly et al., 2015 | All participants were heterosexual (information provided by author). Author re-ran analyses based on whole sample and provided results which we used, so the results do not exactly match results reported in paper (in the paper the authors had omitted a few participants due to incomplete relationship information). |
| Frederick & Jenkins, 2015 | The paper also included dichotomous variables: more than 5 sex partners and more than 14 sex partners. We did not include those (considered redundant) and instead only used the continuous variable number of sex partners. |
| Gallup et al., 2007 | The paper included both SHR circumference and breadth; we only included circumference to keep it consistent with other results. |
| Genovese, 2008 | In the paper, the relationship between HGS and height was reported, but the paper did not include N or information about whether age was controlled for, so first author re-ran analyses. For mesomorphy - offspring number, we assumed N=181 as first author could only find (reliable) data on offspring number for 181 participants in the primary data source. |
| Gettler et al., 2019 | Fertility in The Philippines has now dropped below 3.0 children/woman but was above in 2009 when data was collected (according to https://data.worldbank.org/indicator/SP.DYN.TFRT.IN?locations=PH), therefore coded as a high fertility sample. |
| Hartl et al., 1982 | HGS - offspring number and height - offspring number were also analyzed and reported by Genovese (2008), but that paper did not include N and it was not clear whether age had been controlled for, so first author re-ran analyses. Participants with clearly incomplete or inaccurate family histories were excluded. In cases where family history was clear up to a certain point, or the participant had died, their age at that point was used. Thus, some of these relationships are reported as non-peer reviewed and some as peer-reviewed (the latter in the case where the relationship was reported by Genovese). We did not include general strength, as it was assessed subjectively. |
| Hoppler, Walther et al., 2018 | Ninety-seven percent of sample was white (mentioned in other paper on the same sample). |
| Hönekopp et al., 2007 | Same sample as in Hönekopp et al., 2006, who reported that 80% of the sample were students. |
| Kirchengast, 2000 | Judged to be the same sample as Winkler and Kirchengast, 1994. |
| Kirchengast & Winkler, 1995 | Mean number of children in sample: 1.1 in Rundu and 1.8 in rural areas; however, age range of sample was 18-39 and mean age = 26 so it is young sample with non-completed reproductive histories, therefore coded as a high fertility sample. |
| Klimas et al., 2019 | Ninety-seven percent of sample was white (mentioned in other paper on same sample). Only included men without sexual dysfunction. Excluded participants who had had bleeding or injuries in the mouth in the last few days before testing (clear from other paper on same sample), so we considered blood contamination of saliva sample controlled for. |
| Little et al., 1989 | Population mean and S.D. for height stated in another paper (by the same author); we used that to convert effect sizes. |
| Loehr & O'Hara, 2013 | We assumed it was primarily a white sample. |
| Longman et al., 2018 | Given that this was a young British student sample, we assumed that they were all non-fathers. Baseline testosterone was, in a sense, anticipatory, but we included this paper since effect sizes in this study did not differ substantially from effect sizes in other studies. |
| Lukaszewski et al., 2014 | The first author ran analyses on openly available data. Some extreme outliers in one of the samples (chest strength around ~2, which seems incorrect). However, it made no difference to the results whether they were removed or kept, so we kept them in. |
| Marczak et al., 2018 | 2D:4D was measured directly as well as from digital photos. Not explicit whether that meant that measurement method varied between participants. As we could not be sure that all participants had been measured directly, we coded this as hand scans. |
| Mosing et al., 2015 | Published paper but author submitted results to us. Twin sample; only unrelated individuals included in this sample. We coded it as a heterosexual sample (in the paper, gay participants were excluded), and as a predominantly white sample. |
| Mueller & Mazur, 1997 | N was not explicit in the results, but they stated that 337 participants replied so we assumed N=337. Sample was born 1923-1929 so should have completed most of their reproduction by 1965 when the fertility rate dropped below 3 in the U.S.; therefore coded as a high fertility, industrialized sample. |
| Nagelkerke et al., 2006 | Author sent us raw data. For age at first sexual intercourse, we set cut off at 12 (there were data points <12) as we deemed it unlikely that participants had had sexual onset prior to puberty. |
| Nettle, 2002 | This sample's parents were analyzed in Krzyzanowska et al. (2015) but as the parents reproduced separately and at a different time point compared to this sample, we considered them to be separate samples. |
| Pawlowski et al., 2008 | We assumed this sample was heterosexual. |
| Pawlowski et al., 2000 | Author sent us the results. Coded as a high fertility, industrialized sample. |
| Polo et al., 2019 | The paper also included skeletal muscle mass but this measure was extremely highly correlated with upper-body fat free mass (FFM: *r* = .96, n = 206, *p* < .001) so it was considered redundant to include both predictors, and to be consistent with other studies we kept upper-body FFM. Sample consisted of heterosexual students and non-students: we did not know the proportion of students versus non-students, so we coded it as a non-student sample. Results were considered published as these relationships were reported in paper, although the paper included a type of analyses that we could not use and the authors therefore submitted *r*. |
| Puts et al., 2015 | We assumed the sample were non-fathers. For one sample, testosterone was sampled by saliva between 9AM-1.30PM; we coded that as AM testosterone. |
| Puts et al., 2006 | We judged the sample to be the same one as Putz et al. (2004) and Hodges-Simeon et al. (2011). |
| Putz et al., 2004 | We judged the sample in study 1 to be the same one as Puts et al. (2006) and Hodges-Simeon et al. (2011). |
| Rahman et al., 2005 | Proportion of students vs non-students not clear, so coded as unknown low fertility sample. |
| Rosenfield et al., 2020 | Coded as heterosexual sample as all participants had been married (heterosexually) at some point. |
| Scott & Bajema, 1982 | The paper reported both zero-order correlations and partial correlations controlling for ethnicity. We used zero-order correlations because the sample was from the same group, even if their ethnicities differed. Sample was born 1912-1918 and should therefore have largely completed reproduction by 1965 when the fertility rate dropped below 3 in the U.S.; therefore coded as a high fertility, industrialized sample. |
| Sim & Chun, 2016 | We judged the sample to be the same one as Sim (2013). SHR also reported in Sim (2013); we therefore excluded that paper. |
| Smith et al., 2017 | Same sample/analyses as reported in Apicella et al. (2007) and Apicella (2014); kept those papers as those analyses controlled for age, with the exception of muscle mass/strength - offspring number (also given in Apicella, 2014), as in the latter, N was not explicit. |
| Steiner et al., 2011 | Paper also included SOI and extrapair sexual interest (EPSI), but SOI and EPSI were measured after viewing a video which, for some participants, had sexual content, and those variables were therefore measured after manipulation so we did not include them. Sample consisted of 90% exclusively heterosexual participants, and 10% heterosexual but incidentally gay participants; coded as a heterosexual sample. |
| Stern et al., 2020 | Information about ethnicity not available in paper; however, it was given in other paper on same sample (Kandrik et al., 2016) in which it was reported that 91% of a subsample was white. This sample was therefore coded as predominantly white. |
| Strong et al., 2014 | We excluded 2D:4D following recommendation from author, due to potential measurement issues. |
| Suire et al., 2018 | Baseline recording consisted of just a short utterance repeated after the experimenter, and author therefore suggested to use one of the other recordings (courtship and competition) instead. Results did not show any substantial difference between the recordings, however, so we used baseline to be consistent with other studies. Data set contained virgins: current age set as age at first sexual intercourse, as is commonly done. |
| Tao & Yin, 2016 | Offspring number: S.D. of the mean for the whole sample not given but varied between 1.1-1.3 for each of the three samples so we used that to convert effect sizes (which value was used did not affect the results in any case). We used whole sample rather than the three sub-samples, coded as low fertility. |
| van Anders et al., 2007 | Sampled from a monogamous society, but some of the samples were polyamorous, therefore coded as non-monogamous. |
| van Dongen & Sprengers, 2012 | Sample not specified but we assumed it was from a low fertility, monogamous population. |
| Varella et al., 2014 | Assumed N=80 for all non-significant relationships where N was not specified, as stated elsewhere in the paper. We used results for the two samples combined, not the sub-samples. |
| von Rueden et al., 2010 | DVs not normally distributed but could be transformed to near-normality. |
| Walther et al., 2016 | Ninety-seven percent of sample was white (mentioned in other paper on same sample). |
| Walther et al., 2017b | Ninety-seven percent of sample was white (mentioned in other paper on same sample). |
| Walther et al., 2017c | Ninety-seven percent of sample was white (mentioned in other paper on same sample). |
| Weeden & Sabini, 2007 | Paper also included Sociosexuality measure; however, as we did not know the response scale or direction of responses, we excluded it. |
| Winkler & Kirchengast, 1994 | Judged to be the same sample as Kirchengast, 2000. |

*Note*. 2D:4D = 2^nd^ to 4^th^ finger (digit) ratio; f0 = fundamental frequency, i.e. voice pitch; fWHR = facial width-to-height ratio; HGS = handgrip strength; SHR = shoulder-to-hip ratio; SOI = Sociosexual Orientation Inventory; SOI-R = the Revised Sociosexual Orientation Inventory.

**References**

A. P. Clark, Self-perceived attractiveness and masculinization predict women's sociosexuality. *Evol. Hum. Behav*. 25(2), 113-124 (2004).

C. R. Hodges-Simeon, S. J. Gaulin, D. A. Puts, Voice correlates of mating success in men: examining “contests” versus “mate choice” modes of sexual selection. *Arch. Sex. Behav.* 40(3), 551-557 (2011).

M. Kandrik, A. C. Hahn, C. Han, J. Wincenciak, C. I. Fisher, L. M. DeBruine, B. C. Jones, Does the interaction between cortisol and testosterone predict men’s facial attractiveness?. *Adapt. Hum. Behav. Physiol*. 3(4), 275-281 (2017).

K. Sim, The relationship between sex-typical body shape and quality indicators. *JSEC* 7(2), 97 (2013).
