# Supplementary material for: A meta-analysis of the association between male dimorphism and fitness outcomes in humans
Source: eLife. 2022 Feb 18;11:e65031. doi: 10.7554/eLife.65031 (PMC9106334; doi:10.7554/eLife.65031)
Supplement: Supplementary file 3. [file elife-65031-supp3.docx]

| Supplementary File 3A  *General moderators: for all predictors* | |
| --- | --- |
| Moderator | Description |
| Domain type  Mating measure type | Mating vs reproductive domain.  Mating attitudes (e.g. preferences for short-term relationships/casual sex) vs mating behaviors (e.g. number of sexual partners, age at first sexual intercourse) |
| Reproductive measure type | Fertility (i.e. number of children/grandchildren, age at the birth of the first child) vs reproductive success (i.e. number of surviving children/grandchildren). |
| Sample type | Low fertility samples (i.e. <3.0 children/woman within sample/population at the time of sampling) vs high fertility samples. The latter is considered to correspond to naturally fertile populations. |
| Low fertility samples | Predominantly student samples (i.e. ≥ 50% students) vs mixed/non-student/unknown samples. |
| High fertility samples | Traditional vs industrialized samples. |
| Ethnicity | Predominantly white (i.e. ≥ 75% of sample) vs mixed/non-white /unknown. |
| Marriage system | Monogamy vs non-monogamy/unknown. |
| Publication status | Published vs non-published results. We favored publication status of the relevant *results* rather than of the *paper*, since we retrieved many of our effects from published studies where the key relationship had not been analyzed/was not the focus of the paper. |
| Peer-review | Peer-reviewed vs not peer-reviewed study. |
| Sexual orientation | Heterosexual sample vs gay/mixed/unknown sexual orientation. |
| Normality-transformed variables | Non-transformed vs transformed variables, i.e. whether skewed variables (skew is very common for some of the variables, such as number of sexual partners) had been e.g. log-transformed to normality. |
| Converted effect size | Non-converted vs converted effect sizes, i.e. whether effect size was given as Pearson’s *r* or whether we had used a formula to convert it. The latter results in an estimate of *r*. |
| Age control | Age controlled for in analyses vs not controlled for. We considered age an essential control for all analyses except *i*. where all participants belonged to the same age group, *ii.* for the variables sexual onset/reproductive onset, and *iii.* mating attitudes. |
| Non-relevant controls | No non-relevant vs non-relevant controls included in the analyses. For example, analyses with several non-relevant predictors may produce weaker associations compared to e.g. bivariate correlations with just the relevant predictor and outcome variables. |
| *Note.* We were constrained by information made available in papers. The levels of moderators should therefore be considered to reflect where we knew for certain that a moderator e.g. had been controlled for vs where we could not be certain. For several of our potential moderators, such as the moderators we had selected for voice pitch, not enough papers mentioned having controlled for them and we were therefore unable to analyze those moderators. Additionally, we often did not have enough observations on each level of the moderator variable to be able to run those analyses. This lack of power also prevented us from analyzing combined effects of several moderators; we therefore analyzed moderators one by one. Moderators were coded into two levels wherever possible, as otherwise we would often have had too few observations/level to be able to run the analysis. It should also be noted that some moderators are likely confounded; for example, non-monogamous populations are almost always high fertility, traditional populations. | |

| Supplementary File 3B | |
| --- | --- |
| *Facial masculinity moderators* | |
| Moderator | Description |
| Measurement type | Objectively measured masculinity (using geometric morphometric analyses) vs observer-rated masculinity vs fWHR (i.e. facial width-to-height ratio). |
| Standardization of photographs | Photographs taken under standardized vs not standardized/semi-standardized/unknown conditions. |
| Angle of photographs | Front-facing vs not front-facing/unknown angle of photographs. |
| Masked photographs | Masked vs not masked photographs/unknown. Only coded for rated facial masculinity. |
| Adiposity | Adiposity/body mass index (BMI) controlled for vs not controlled for/unknown. Only coded for rated facial masculinity. |
| Color vs black & white photographs | Color vs black & white photographs/unknown. Only coded for rated facial masculinity. |
| Facial expression | Neutral vs smiling/mixed/unknown facial expressions. Only coded for rated facial masculinity. |
| Facial hair | Clean-shaven vs not clean-shaven/mixed/unknown. Only coded for rated facial masculinity. |
| *Note.* Moderators were coded into two levels wherever possible, as otherwise we would often have had too few observations/level to be able to run the analysis. | |

Supplementary File 3C

|  | |
| --- | --- |
| *Body masculinity moderators* | |
| Moderator | Description |
| Number of measurements | Only coded for measured body masculinity. Typically referred to repeat measurements but in some cases, different measurements were used. |
| Adiposity | Adiposity/BMI controlled for vs not controlled for/unknown. Only coded for rated body masculinity. |
| Measurement type | Measured vs observer- or own-rated body masculinity. Measured body masculinity included e.g. strength, circumference of shoulder-to-hip ratio, and bioelectrical measurement of fat-free mass. |
| Body masculinity type | Strength vs body shape vs muscle mass. Strength was typically assessed through measured handgrip strength. Body shape included body measurements (see measurement type above) and rated body masculinity. Muscle mass was measured (see above) or rated. |
| *Note.* Moderators were coded into two levels wherever possible, as otherwise we would often have had too few observations/level to be able to run the analysis. | |

Supplementary File 3D

|  | |
| --- | --- |
| *2D:4D moderators* | |
| Moderator | Description |
| Measurement type | Measured directly vs measured from hand scans/photographs vs self-reported vs unknown. |
| Number of measurements | Only coded for experimenter-measured (directly or from hand scans). |
| Finger injuries | Controlled for vs not controlled for. |
| Left vs right | Left vs right hand 2D:4D. 2D:4D dimorphism is typically claimed to be more pronounced in the right hand (Hönekopp et al., 2006). |
| *Note.* Moderators were coded into two levels wherever possible, as otherwise we would often have had too few observations/level to be able to run the analysis. | |

Supplementary File 3E

|  | |
| --- | --- |
| *Voice pitch moderators* | |
| Moderator | Description |
| Sex of experimenter | Female vs male vs unknown. |
| Illness | Illnesses (colds etc. that could influence voice pitch) controlled for/excluded vs not. |
| Smoker | Smoking participants controlled for/excluded vs not. |
| Condition | Baseline vs courtship cs competitive type of recording. |
| *Note.* Based on information available in the papers, none of these potential moderators had been controlled for in any of the studies. | |

Supplementary File 3F

|  | |
| --- | --- |
| *Height moderators* | |
| Moderator | Description |
| Measurement type | Experimenter-measured vs self-reported vs unknown. |
| Number of measurements | Only coded for experimenter-measured height. |
| *Note.* Moderators were coded into two levels wherever possible, as otherwise we would often have had too few observations/level to be able to run the analysis. | |

Supplementary File 3G

|  | |
| --- | --- |
| *Testosterone levels moderators* | |
| Moderator | Description |
| How assayed | Assayed from blood vs saliva vs unknown. |
| Time of day | Assayed in the AM vs PM vs unknown. |
| Blood contamination | Checked for vs not checked for/unknown. Only coded for saliva assayed T levels. |
| Fatherhood | Non-fathers vs fathers vs mixed/unknown |
| Relationship status | Married/in committed relationship vs single vs mixed/unknown. |
| *Note.* Moderators were coded into two levels wherever possible, as otherwise we would often have had too few observations/level to be able to run the analysis. | |
