# Supplementary material for: A meta-analysis of the association between male dimorphism and fitness outcomes in humans
Source: eLife. 2022 Feb 18;11:e65031. doi: 10.7554/eLife.65031 (PMC9106334; doi:10.7554/eLife.65031)
Supplement: Supplementary file 4. [file elife-65031-supp4.docx]

Supplementary File 4A

| *Facial masculinity: moderation analyses. The intercept shows the 'simple effect' for the reference category (specified) and the moderator effect shows the change in effect size for that category relative to the reference category. Moderators are bolded if significant after controlling for multiple comparisons, as indicated by computation of q-values. The full list of q-values can be found in Supplementary File 7.* | | | | | | |
| --- | --- | --- | --- | --- | --- | --- |
| \| Mating vs reproductive domain \| \| \| \| \| \| \| \| --- \| --- \| --- \| --- \| --- \| --- \| --- \| \|  \|  \| B \| SE \| [95% CI] \| *z* \| *p* \| \| Domain type \| Intercept (mating domain) \| 0.088 \| 0.042 \| 0.007, 0.169 \| 2.120 \| .034 \| \|  \| Reproductive domain \| 0.006 \| 0.072 \| -0.135, 0.148 \| 0.089 \| .929 \| | | | | | | |
| Mating domain (MAT), mating behaviors & mating attitudes | | | | | | |
|  |  | B | SE | [95% CI] | *z* | *p* |
| MAT measure type | Intercept (MAT behaviors) | 0.047 | 0.041 | -0.033, 0.128 | 1.150 | .250 |
|  | MAT attitudes | 0.038 | 0.061 | -0.082, 0.159 | 0.622 | .534 |
| Ethnicity | Intercept (predominantly white) | 0.114 | 0.057 | 0.003, 0.225 | 2.007 | .045 |
|  | Mixed/other/unknown | -0.075 | 0.084 | -0.241, 0.090 | -0.892 | .373 |
| Publication status | Intercept (published results) | 0.046 | 0.058 | -0.067, 0.159 | 0.801 | .423 |
|  | Non-published results | 0.092 | 0.093 | -0.090, 0.273 | 0.990 | .322 |
| Publication status: MAT behaviors | Intercept (published results) | 0.016 | 0.056 | -0.094, 0.126 | 0.281 | .778 |
|  | Non-published results | 0.023 | 0.098 | -0.169, 0.215 | 0.233 | .816 |
| Sexual orientation | Intercept (heterosexual sample) | 0.044 | 0.055 | -0.063, 0.151 | 0.806 | .420 |
|  | Gay/mixed/unknown | 0.105 | 0.092 | -0.075, 0.285 | 1.145 | .252 |
| Age control | Intercept (age controlled for) | 0.065 | 0.060 | -0.052, 0.181 | 1.086 | .277 |
|  | Age not controlled for | 0.038 | 0.098 | -0.154, 0.230 | 0.387 | .699 |
| Age control: MAT behaviors | Intercept (age controlled for) | 0.017 | 0.052 | -0.086, 0.119 | 0.318 | .750 |
|  | Age not controlled for | 0.024 | 0.100 | -0.172, 0.220 | 0.241 | .810 |
| Measurement type | Intercept (measured) | 0.105 | 0.058 | -0.008, 0.219 | 1.825 | .068 |
|  | Rated | -0.009 | 0.079 | -0.162, 0.146 | -0.108 | .914 |
|  | fWHR: *s* =2 |  |  |  |  |  |
| Other moderators with too few *k*/*s*: | | | | | | |
| Sample type: low vs high fertility; Low fertility samples: predominantly students vs non-students; High fertility samples: traditional vs industrialized; Marriage system: monogamy vs non-monogamy; Peer-reviewed vs not peer-reviewed; Normality-transformed variables; Converted effect size; Non-relevant controls, Standardization of photographs; Angle of photographs; Masked photographs; Adiposity; Color vs black & white photographs; Facial expression; Facial hair. | | | | | | |
| Reproductive domain (REP), fertility & reproductive success | | | | | | |
| Moderators with too few *k*/*s*: | | | | | | |
| REP measure type: reproductive success vs fertility; Sample type: low vs high fertility; Low fertility samples: predominantly students vs non-students; High fertility samples: traditional vs industrialized; Ethnicity: predominantly white vs not; Marriage system: monogamy vs non-monogamy; Publication status: published results; Peer-reviewed; Sexual orientation: heterosexual sample vs gay/mixed/unknown; Normality-transformed variables; Converted effect size; Age controlled for; Non-relevant controls, Measurement type: measured vs rated vs fWHR; Standardization of photographs; Angle of photographs; Masked photographs; Adiposity; Color vs black & white photographs; Facial expression; Facial hair. | | | | | | |
| *Note.* *k* = number of observations, MAT = mating, REP = reproductive, *s* = number of samples. Moderation analyses were only run where each level of the moderator included observations from at least two studies and three independent samples. Analyses were run on the mating measures mating behaviors and mating attitudes, and the reproductive measures fertility and reproductive success when there were enough observations to do so. | | | | | | |

Supplementary File 4B

|  | | | | | | | |
| --- | --- | --- | --- | --- | --- | --- | --- |
| *Body* *masculinity: moderation analyses. The intercept shows the 'simple effect' for the reference category (specified) and the moderator effect shows the change in effect size for that category relative to the reference category. Moderators are bolded if significant after controlling for multiple comparisons, as indicated by computation of q-values. The full list of q-values can be found in Supplementary File 7.* | | | | | | | |
| \| Mating vs reproductive domain \| \| \| \| \| \| \| \| --- \| --- \| --- \| --- \| --- \| --- \| --- \| \|  \|  \| B \| SE \| [95% CI] \| *z* \| *p* \| \| Domain type \| Intercept (mating domain) \| 0.132 \| 0.021 \| 0.092, 0.173 \| 6.414 \| <.001 \| \|  \| Reproductive domain \| 0.019 \| 0.042 \| -0.064, 0.102 \| 0.439 \| .661 \| | | | | | | |  |
| Mating domain (MAT), mating behaviors & mating attitudes | | | | | | | |
|  |  | B | SE | [95% CI] | *z* | *p* | |
| MAT measure type | Intercept (MAT behaviors) | 0.139 | 0.022 | 0.096, 0.181 | 6.382 | <.001 | |
|  | MAT attitudes | -0.024 | 0.031 | -0.085, 0.037 | -0.773 | .440 | |
| Sample type | Intercept (low fertility) | 0.136 | 0.023 | 0.091, 0.181 | 5.878 | <.001 | |
|  | High fertility | -0.024 | 0.078 | -0.177, 0.129 | -0.307 | .759 | |
| Sample type: MAT behaviors | Intercept (low fertility) | 0.147 | 0.024 | 0.099, 0.194 | 6.019 | <.001 | |
|  | High fertility | -0.035 | 0.078 | -0.188, 0.119 | -0.443 | .658 | |
| Low fertility sample | Intercept (predominantly students) | 0.157 | 0.023 | 0.111, 0.203 | 6.699 | <.001 | |
|  | Non-students/ mixed/unknown | -0.118 | 0.051 | -0.218, -0.019 | -2.336 | .020 | |
| Low fertility sample: MAT behaviors | Intercept (predominantly students) | 0.172 | 0.024 | 0.125, 0.218 | 7.285 | <.001 | |
|  | **Non-students/ mixed/unknown** | **-0.128** | **0.049** | **-0.224, -0.033** | **-2.632** | **.009** | |
| Ethnicity | Intercept (predominantly white) | 0.116 | 0.044 | 0.030, 0.203 | 2.643 | .008 | |
|  | Mixed/other/unknown | 0.024 | 0.051 | -0.076, 0.124 | 0.464 | .643 | |
| Ethnicity: MAT behaviors | Intercept (predominantly white) | 0.116 | 0.044 | 0.031, 0.202 | 2.661 | .008 | |
|  | Mixed/other/unknown | 0.038 | 0.052 | -0.064, 0.139 | 0.728 | .467 | |
| Marriage system | Intercept (monogamy) | 0.139 | 0.022 | 0.095, 0.182 | 6.230 | <.001 | |
|  | Non-monogamy | -0.095 | 0.096 | -0.283, 0.093 | -0.991 | .322 | |
| Marriage system: MAT behaviors | Intercept (monogamy) | 0.149 | 0.023 | 0.104, 0.195 | 6.422 | <.001 | |
|  | Non-monogamy | -0.106 | 0.096 | -0.294, 0.082 | -1.104 | .270 | |
| Publication status | Intercept (published results) | 0.167 | 0.026 | 0.117, 0.218 | 6.470 | <.001 | |
|  | Non-published results | -0.086 | 0.039 | -0.163, -0.009 | -2.181 | .029 | |
| Publication status: MAT attitudes | Intercept (published results) | 0.099 | 0.044 | 0.013, 0.184 | 2.251 | .024 | |
|  | Non-published results | -0.079 | 0.081 | -0.237, 0.079 | -0.975 | .330 | |
| Publication status: MAT behaviors | Intercept (published results) | 0.177 | 0.028 | 0.123, 0.231 | 6.402 | <.001 | |
|  | Non-published results | -0.087 | 0.044 | -0.172, -0.001 | -1.978 | .048 | |
| Peer-review | Intercept (peer-reviewed) | 0.136 | 0.024 | 0.088, 0.184 | 5.576 | <.001 | |
|  | Not peer-reviewed | -0.012 | 0.058 | -0.126, 0.102 | -0.204 | .838 | |
| Peer-review: MAT behaviors | Intercept (peer-reviewed) | 0.142 | 0.025 | 0.093, 0.192 | 5.636 | <.001 | |
|  | Not peer-reviewed | 0.005 | 0.063 | -0.118, 0.128 | 0.085 | .933 | |
| Sexual orientation | Intercept (heterosexual sample) | 0.177 | 0.030 | 0.118, 0.235 | 5.948 | <.001 | |
|  | Gay/mixed/unknown | -0.085 | 0.041 | -0.165, -0.006 | -2.098 | .036 | |
| Sexual orientation: MAT attitudes | Intercept (heterosexual sample) | 0.045 | 0.057 | -0.067, 0.157 | 0.781 | .435 | |
|  | Gay/mixed/unknown | 0.062 | 0.077 | -0.089, 0.212 | 0.804 | .421 | |
| Sexual orientation: MAT behaviors | Intercept (heterosexual sample) | 0.188 | 0.031 | 0.127, 0.249 | 6.069 | <.001 | |
|  | Gay/mixed/unknown | -0.088 | 0.042 | -0.171, -0.006 | -2.091 | .037 | |
| Normality-transformed variables | Intercept (non-transformed variables) | 0.137 | 0.025 | 0.088, 0.185 | 5.523 | <.001 | |
|  | Transformed variables | 0.038 | 0.047 | -0.054, 0.129 | 0.810 | .418 | |
| Normality-transformed variables: MAT behaviors | Intercept (non-transformed variables) | 0.049 | 0.037 | -0.024, 0.122 | 1.321 | .186 | |
|  | **Transformed variables** | **0.165** | **0.054** | **0.060, 0.270** | **3.091** | **.002** | |
| Converted effect size | Intercept (not converted) | 0.144 | 0.026 | 0.093, 0.194 | 5.587 | <.001 | |
|  | Converted | 0.003 | 0.063 | -0.121, 0.128 | 0.053 | .958 | |
| Converted effect size: MAT behaviors | Intercept (not converted) | 0.152 | 0.028 | 0.098, 0.206 | 5.508 | <.001 | |
|  | Converted | -0.005 | 0.065 | -0.133, 0.123 | -0.076 | .940 | |
| Age control | Intercept (age controlled for) | 0.098 | 0.031 | 0.037, 0.158 | 3.147 | .002 | |
|  | **Age not controlled for** | **0.103** | **0.042** | **0.020, 0.186** | **2.441** | **.015** | |
| Age control: MAT behaviors | Intercept (age controlled for) | 0.107 | 0.033 | 0.043, 0.171 | 3.277 | .001 | |
|  | Age not controlled for | 0.096 | 0.045 | 0.009, 0.183 | 2.153 | .031 | |
| Number of measurements | Intercept (unknown number of measurements) | 0.076 | 0.041 | -0.005, 0.156 | 1.851 | .064 | |
|  | 2 measurements | 0.046 | 0.047 | -0.046, 0.137 | 0.974 | .330 | |
|  | 3 measurements | 0.126 | 0.062 | 0.004, 0.247 | 2.025 | .043 | |
|  | 1 measurement: *s* = 1 |  |  |  |  |  | |
| Number of measurements: MAT attitudes | Intercept (unknown number of measurements) | 0.117 | 0.054 | 0.010, 0.223 | 2.144 | .032 | |
|  | 2 measurements | -0.070 | 0.071 | -0.210, 0.069 | -0.987 | .324 | |
|  | 1 measurement: *s* = 1 |  |  |  |  |  | |
|  | 3 measurements: *s* = 0 |  |  |  |  |  | |
| Number of measurements: MAT behaviors | Intercept (unknown number of measurements) | 0.061 | 0.048 | -0.034, 0.155 | 1.259 | .208 | |
|  | 2 measurements | 0.093 | 0.059 | -0.023, 0.210 | 1.567 | .117 | |
|  | 3 measurements | 0.151 | 0.070 | 0.015, 0.288 | 2.169 | .030 | |
|  | 1 measurement: *s* = 1 |  |  |  |  |  | |
| Measurement type | Intercept (measured) | 0.081 | 0.040 | 0.002, 0.159 | 2.011 | .044 | |
|  | **Rated** | **0.177** | **0.066** | **0.048, 0.306** | **2.695** | **.007** | |
| Measurement type: MAT behaviors | Intercept (measured) | 0.087 | 0.041 | 0.007, 0.167 | 2.121 | .034 | |
|  | **Rated** | **0.174** | **0.066** | **0.044, 0.303** | **2.630** | **.009** | |
| Body masculinity type | Intercept (strength) | 0.187 | 0.031 | 0.126, 0.248 | 5.974 | <.001 | |
|  | **Body shape** | **-0.099** | **0.034** | **-0.165, -0.033** | **-2.945** | **.003** | |
|  | Muscle mass | -0.108 | 0.071 | -0.247, 0.031 | -1.529 | .126 | |
| Body masculinity type: MAT behaviors | Intercept (strength) | 0.205 | 0.035 | 0.136, 0.274 | 5.8130 | <.001 | |
|  | **Body shape** | **-0.105** | **0.040** | **-0.184, -0.026** | **-2.615** | **.009** | |
|  | Muscle mass | -0.124 | 0.074 | -0.269, 0.021 | -1.676 | .094 | |
| Other moderators with too few *k*/*s*: | | | | | | | |
| High fertility sample: traditional vs industrialized; Non-relevant controls, Adiposity. | | | | | | | |
| Reproductive domain (REP), fertility & reproductive success | | | | | | | |
|  |  | B | SE | [95% CI] | *z* | *p* | |
| REP measure type | Intercept (Reproductive success) | 0.170 | 0.071 | 0.032, 0.309 | 2.417 | .016 | |
|  | Fertility | -0.034 | 0.081 | -0.192, 0.125 | -0.417 | .677 | |
| Publication status | Intercept (published results) | 0.222 | 0.065 | 0.095, 0.349 | 3.418 | .001 | |
|  | Non-published results | -0.107 | 0.075 | -0.254, 0.040 | -1.423 | .155 | |
| Normality-transformed variables | Intercept (non-transformed variables) | 0.136 | 0.046 | 0.045, 0.227 | 2.928 | .003 | |
|  | Transformed variables | 0.022 | 0.071 | -0.117, 0.161 | 0.311 | .756 | |
| Normality-transformed variables: Fertility | Intercept (non-transformed variables) | 0.095 | 0.042 | 0.012, 0.178 | 2.246 | .025 | |
|  | Transformed variables | 0.078 | 0.063 | -0.045, 0.200 | 1.239 | .215 | |
| Converted effect size | Intercept (not converted) | 0.089 | 0.034 | 0.023, 0.155 | 2.635 | .008 | |
|  | **Converted** | **0.143** | **0.059** | **0.028, 0.258** | **2.437** | **.015** | |
| Non-relevant controls | Intercept (no non-relevant controls) | 0.136 | 0.042 | 0.054, 0.219 | 3.232 | .001 | |
|  | Non-relevant controls | 0.032 | 0.082 | -0.128, 0.193 | 0.395 | .693 | |
| Number of measurements | Intercept (unknown number of measurement) | 0.117 | 0.055 | 0.010, 0.224 | 2.138 | .033 | |
|  | 3 measurements | 0.067 | 0.084 | -0.098, 0.232 | 0.797 | .426 | |
|  | 1 measurement: *s* = 0 |  |  |  |  |  | |
|  | 2 measurements: *s* = 0 |  |  |  |  |  | |
| Number of measurements: Reproductive success | Intercept (unknown number of measurement) | 0.072 | 0.201 | -0.323, 0.466 | 0.356 | .722 | |
|  | 3 measurements | 0.136 | 0.178 | -0.213, 0.485 | 0.763 | .446 | |
|  | 1 measurement: *s* = 0 |  |  |  |  |  | |
|  | 2 measurements: *s* = 0 |  |  |  |  |  | |
| Body masculinity type | Intercept (strength) | 0.112 | 0.036 | 0.041, 0.183 | 3.108 | .002 | |
|  | Muscle mass | 0.028 | 0.066 | -0.101, 0.158 | 0.430 | .667 | |
|  | Body shape: *s* = 0 |  |  |  |  |  | |
| Other moderators with too few *k*/*s*: | | | | | | | |
| Sample type: low vs high fertility; Low fertility sample: students vs non-students; High fertility sample: traditional vs industrialized; Ethnicity: predominantly white vs not; Marriage system: monogamy vs non-monogamy; Peer-reviewed vs not peer-reviewed; Sexual orientation: heterosexual sample vs gay/mixed/unknown; Age controlled for; Adiposity; Measurement type: measured vs rated. | | | | | | | |
| *Note.* *k* = number of observations, MAT = mating, REP = reproductive, *s* = number of samples. Moderation analyses were only run where each level of the moderator included observations from at least two studies and three independent samples. Analyses were run on the mating measures mating behaviors and mating attitudes, and the reproductive measures fertility and reproductive success when there were enough observations to do so. | | | | | | | |

Supplementary File 4C

| *2D:4D: moderation analyses. The intercept shows the 'simple effect' for the reference category (specified) and the moderator effect shows the change in effect size for that category relative to the reference category. Moderators are bolded if significant after controlling for multiple comparisons, as indicated by computation of q-values. The full list of q-values can be found in Supplementary File 7.* | | | | | | | | | | | | | | |
| --- | --- | --- | --- | --- | --- | --- | --- | --- | --- | --- | --- | --- | --- | --- |
| Mating vs reproductive domain | | | | | | | | | | | | |  |  |
|  | |  | | B | | SE | [95% CI] | | | *z* | | *p* |  |  |
| Domain type | | Intercept (mating domain) | | 0.050 | | 0.018 | 0.015, 0.084 | | | 2.809 | | .005 |  |  |
|  | | Reproductive domain | | 0.007 | | 0.004 | 0.000, 0.014 | | | 1.997 | | .046 |  |  |
| Mating domain (MAT), mating behaviors & mating attitudes | | | | | | | | | | | | | | |
|  | |  | | B | | SE | | | [95% CI] | *z* | | *p* | | |
| MAT measure type | | Intercept (MAT behaviors) | | 0.042 | | 0.022 | | | -0.002, 0.085 | 1.870 | | .062 | | |
|  | | MAT attitudes | | 0.004 | | 0.039 | | | -0.072, 0.080 | 0.097 | | .923 | | |
| Low fertility sample | | Intercept (predominantly students) | | 0.036 | | 0.022 | | | -0.007, 0.078 | 1.646 | | .100 | | |
|  | | Non-students/mixed/unknown | | 0.014 | | 0.042 | | | -0.069, 0.096 | 0.321 | | .749 | | |
| Low fertility sample:  MAT behaviors | | Intercept (predominantly students) | | 0.033 | | 0.023 | | | -0.012, 0.077 | 1.419 | | .156 | | |
|  | | Non-students/mixed/unknown | | 0.053 | | 0.049 | | | -0.042, 0.148 | 1.096 | | .273 | | |
| Ethnicity | | Intercept (predominantly white) | | 0.072 | | 0.022 | | | 0.030, 0.115 | 3.333 | | .001 | | |
|  | | **Mixed/other/unknown** | | **-0.080** | | **0.032** | | | **-0.143, -0.016** | **-2.462** | | **.014** | | |
| Ethnicity:  MAT attitudes | | Intercept (predominantly white) | | 0.113 | | 0.063 | | | -0.010, 0.236 | 1.809 | | .071 | | |
|  | | Mixed/other/unknown | | -0.128 | | 0.081 | | | -0.287, 0.032 | -1.572 | | .116 | | |
| Ethnicity:  MAT behaviors | | Intercept (predominantly white) | | 0.085 | | 0.026 | | | 0.034, 0.137 | 3.245 | | .001 | | |
|  | | **Mixed/other/unknown** | | **-0.088** | | **0.036** | | | **-0.158, -0.017** | **-2.423** | | **.015** | | |
| Publication status | | Intercept (published results) | | 0.042 | | 0.022 | | | -0.000, 0.085 | 1.954 | | .051 | | |
|  | | Non-published results | | -0.020 | | 0.040 | | | -0.098, 0.059 | -0.492 | | .623 | | |
| Publication status:  MAT behaviors | | Intercept (published results) | | 0.046 | | 0.027 | | | -0.006, 0.098 | 1.719 | | .086 | | |
|  | | Non-published results | | -0.017 | | 0.044 | | | -0.103, 0.068 | -0.396 | | .692 | | |
| Peer-review | | Intercept (peer-reviewed) | | 0.031 | | 0.019 | | | -0.007, 0.068 | 1.615 | | .106 | | |
|  | | Not peer-reviewed | | 0.034 | | 0.057 | | | -0.078, 0.146 | 0.592 | | .554 | | |
| Peer-review: MAT behaviors | | Intercept (peer-reviewed) | | 0.029 | | 0.021 | | | -0.013, 0.071 | 1.349 | | .178 | | |
|  | | Not peer-reviewed | | 0.070 | | 0.063 | | | -0.054, 0.193 | 1.103 | | .270 | | |
| Sexual orientation | | Intercept (heterosexual sample) | | 0.014 | | 0.026 | | | -0.038, 0.065 | 0.520 | | .603 | | |
|  | | Gay/mixed/unknown | | 0.035 | | 0.034 | | | -0.031, 0.102 | 1.041 | | .298 | | |
| Sexual orientation: MAT behaviors | | Intercept (heterosexual sample) | | 0.024 | | 0.033 | | | -0.041, 0.089 | 0.731 | | .465 | | |
|  | | Gay/mixed/unknown | | 0.024 | | 0.042 | | | -0.058, 0.105 | 0.566 | | .571 | | |
| Normality-transformed variables | | Intercept (non-transformed variables) | | 0.011 | | 0.026 | | | -0.039, 0.061 | 0.443 | | .658 | | |
|  | | **Transformed variables** | | **0.103** | | **0.043** | | | **0.020, 0.187** | **2.416** | | **.016** | | |
| Normality-transformed variables: MAT behaviors | | Intercept (non-transformed variables) | | 0.009 | | 0.026 | | | -0.041, 0.060 | 0.368 | | .713 | | |
|  | | **Transformed variables** | | **0.102** | | **0.040** | | | **0.024, 0.180** | **2.553** | | **.011** | | |
| Age control | | Intercept (age controlled for) | | 0.069 | | 0.051 | | | -0.031, 0.169 | 1.358 | | .175 | | |
|  | | Age not controlled for | | -0.033 | | 0.057 | | | -0.144, 0.078 | -0.583 | | .560 | | |
| Age control: MAT behaviors | | Intercept (age controlled for) | | 0.077 | | 0.056 | | | -0.032, 0.186 | 1.387 | | .165 | | |
|  | | Age not controlled for | | -0.025 | | 0.063 | | | -0.148, 0.098 | -0.401 | | .689 | | |
| Number of measurements | | Intercept (unknown number of measurements) | | 0.021 | | 0.021 | | | -0.020, 0.061 | 1.009 | | .313 | | |
|  | | 2 measurements | | -0.023 | | 0.030 | | | -0.082, 0.035 | -0.782 | | .434 | | |
|  | | **3 measurements** | | **0.102** | | **0.037** | | | **0.030, 0.175** | **2.759** | | **.006** | | |
|  | | 1 measurement: *s* = 1 | |  | |  | | |  |  | |  | | |
| Number of measurements: MAT behaviors | | Intercept (unknown number of measurements) | | 0.021 | | 0.021 | | | -0.020, 0.061 | 1.009 | | .313 | | |
|  | | 2 measurements | | -0.023 | | 0.030 | | | -0.082, 0.035 | -0.782 | | .434 | | |
|  | | **3 measurements** | | **0.102** | | **0.037** | | | **0.030, 0.175** | **2.759** | | **.006** | | |
|  | | 1 measurement: *s* = 1 | |  | |  | | |  |  | |  | | |
| Measurement type | | Intercept (directly) | | 0.004 | | 0.032 | | | -0.058, 0.066 | 0.128 | | .898 | | |
|  | | Hand scans | | 0.091 | | 0.042 | | | 0.008, 0.174 | 2.145 | | .032 | | |
|  | | Unknown | | 0.005 | | 0.048 | | | -0.090, 0.098 | 0.093 | | .926 | | |
|  | | Self-reported: *s* = 1 | |  | |  | | |  |  | |  | | |
| Measurement type:  MAT behaviors | | Intercept (directly) | | 0.005 | | 0.032 | | | -0.059, 0.069 | 0.152 | | .879 | | |
|  | | Hand scans | | 0.083 | | 0.043 | | | -0.002, 0.168 | 1.913 | | .056 | | |
|  | | Unknown | | 0.000 | | 0.049 | | | -0.095, 0.096 | 0.006 | | .995 | | |
|  | | Self-reported: *s* = 0 | |  | |  | | |  |  | |  | | |
| Finger injuries | | Intercept (finger injuries controlled for) | | -0.002 | | 0.046 | | | -0.093, 0.088 | -0.048 | | .962 | | |
|  | | Finger injuries not controlled for | | 0.046 | | 0.050 | | | -0.053, 0.144 | 0.908 | | .364 | | |
| Finger injuries: MAT behaviors | | Intercept (finger injuries controlled for) | | 0.016 | | 0.047 | | | -0.077, 0.109 | 0.335 | | .738 | | |
|  | | Finger injuries not controlled for | | 0.030 | | 0.053 | | | -0.074, 0.134 | 0.566 | | .572 | | |
| Left vs right hand ratios | | Intercept (right 2D:4D) | | 0.039 | | 0.021 | | | -0.002, 0.080 | 1.872 | | .061 | | |
|  | | Left 2D:4D | | -0.002 | | 0.005 | | | -0.013, 0.009 | -0.363 | | .717 | | |
| Left vs right hand ratios: MAT attitudes | | Intercept (right 2D:4D) | | 0.006 | | 0.066 | | | -0.124, 0.136 | 0.093 | | .926 | | |
|  | | Left 2D:4D | | 0.043 | | 0.060 | | | -0.076, 0.161 | 0.707 | | .479 | | |
| Left vs right hand ratios: MAT behaviors | | Intercept (right 2D:4D) | | 0.037 | | 0.029 | | | -0.021, 0.094 | 1.253 | | .210 | | |
|  | | Left 2D:4D | | 0.014 | | 0.032 | | | -0.048, 0.077 | 0.449 | | .653 | | |
| Other moderators with too few *k*/*s*: | | | | | | | | | | | | | | |
| Sample type: low vs high fertility; High fertility sample: traditional vs industrialized; Marriage system: monogamy vs non-monogamy; Converted effect size; Non-relevant controls. | | | | | | | | | | | | | | |
| Reproductive domain (REP), fertility & reproductive success | | | | | | | | | | | | | | |
|  | |  | | B | | SE | | | [95% CI] | *z* | | *p* | | |
| REP measure type | | Intercept (reproductive success) | | 0.170 | | 0.066 | | | 0.042, 0.301 | 2.592 | | .010 | | |
|  | | Fertility | | -0.135 | | 0.078 | | | -0.289, 0.016 | -1.751 | | .080 | | |
| Sample type | | Intercept (low fertility) | | 0.075 | | 0.057 | | | -0.037, 0.187 | 1.319 | | .187 | | |
|  | | High fertility | | 0.002 | | 0.072 | | | -0.143, 0.140 | -0.022 | | .983 | | |
| High fertility sample | | Intercept (traditional) | | 0.028 | | 0.089 | | | -0.145, 0.202 | 0.320 | | .749 | | |
|  | | Industrialized | | 0.110 | | 0.126 | | | -0.137, 0.357 | 0.874 | | .382 | | |
| Ethnicity | | Intercept (predominantly white) | | 0.066 | | 0.055 | | | -0.042, 0.175 | 1.196 | | .232 | | |
|  | | Mixed/other/unknown | | 0.016 | | 0.072 | | | -0.124, 0.157 | 0.227 | | .821 | | |
| Marriage system | | Intercept (monogamy) | | 0.054 | | 0.042 | | | -0.027, 0.136 | 1.304 | | .192 | | |
|  | | Non-monogamy | | 0.116 | | 0.089 | | | -0.058, 0.290 | 1.306 | | .192 | | |
| Publication status | | Intercept (published results) | | 0.105 | | 0.040 | | | 0.026, 0.184 | 2.601 | | .009 | | |
|  | | Non-published results | | -0.166 | | 0.093 | | | -0.348, 0.015 | -1.795 | | .073 | | |
| Sexual orientation | | Intercept (heterosexual sample) | | 0.039 | | 0.066 | | | -0.091, 0.169 | 0.584 | | .560 | | |
|  | | Gay/mixed/unknown | | 0.062 | | 0.087 | | | -0.109, 0.233 | 0.707 | | .480 | | |
| Sexual orientation: Fertility | | Intercept (heterosexual sample) | | 0.037 | | 0.079 | | | -0.119, 0.192 | 0.462 | | .644 | | |
|  | | Gay/mixed/unknown | | -0.019 | | 0.125 | | | -0.264, 0.225 | -0.155 | | .877 | | |
| Normality-transformed variables | | Intercept (non-transformed variables) | | 0.031 | | 0.036 | | | -0.040, 0.101 | 0.851 | | .395 | | |
|  | | Transformed variables | | 0.138 | | 0.067 | | | 0.006, 0.269 | 2.054 | | .040 | | |
| Age control | | Intercept (age controlled for) | | 0.070 | | 0.037 | | | -0.002, 0.142 | 1.915 | | .056 | | |
|  | | Age not controlled for | | 0.077 | | 0.085 | | | -0.088, 0.243 | 0.916 | | .360 | | |
| Measurement type | | Intercept (directly) | | 0.072 | | 0.064 | | | -0.053, 0.197 | 1.133 | | .257 | | |
|  | | Hand scans | | 0.053 | | 0.106 | | | -0.155, 0.261 | 0.502 | | .616 | | |
|  | | Self-reported: *s* = 1 | |  | |  | | |  |  | |  | | |
|  | | Unknown: *s* = 0 | |  | |  | | |  |  | |  | | |
| Finger injuries | | Intercept (finger injuries controlled for) | | 0.136 | | 0.062 | | | 0.014, 0.257 | 2.190 | | .029 | | |
|  | | Finger injuries not controlled for | | -0.104 | | 0.081 | | | -0.262, -0.054 | -1.292 | | .196 | | |
| Left vs right hand ratios | | Intercept (right 2D:4D) | | 0.070 | | 0.044 | | | -0.017, 0.156 | 1.584 | | .113 | | |
|  | | Left 2D:4D | | 0.004 | | 0.004 | | | -0.005, 0.012 | 0.840 | | .401 | | |
| Left vs right hand ratios: Fertility | | Intercept (right 2D:4D) | | 0.031 | | 0.050 | | | -0.067, 0.128 | 0.618 | | .537 | | |
|  | | Left 2D:4D | | 0.004 | | 0.004 | | | -0.005, 0.012 | 0.853 | | .394 | | |
| Other moderators with too few *k*/*s*: | | | | | | | | | | | | | | |
| Low fertility sample: students vs non-students; Peer-reviewed vs not peer-reviewed; Converted effect size; Non-relevant controls; Number of measurements; Left vs right hand. | | | | | | | | | | | | | | |
| *Note.* *k* = number of observations, MAT = mating, REP = reproductive, *s* = number of samples. Moderation analyses were only run where each level of the moderator included observations from at least two studies and three independent samples. Analyses were run on the mating measures mating behaviors and mating attitudes, and the reproductive measures fertility and reproductive success when there were enough observations to do so. | | | | | | | | | | | | | | |

Supplementary File 4D

|  | | | | | | | | |
| --- | --- | --- | --- | --- | --- | --- | --- | --- |
| *Voice pitch: moderation analyses. The intercept shows the 'simple effect' for the reference category (specified) and the moderator effect shows the change in effect size for that category relative to the reference category. Moderators are bolded if significant after controlling for multiple comparisons, as indicated by computation of q-values. The full list of q-values can be found in Supplementary File 7.* | | | | | | | | |
| Mating vs reproductive domain | | | | | | |  |  |
|  |  | B | SE | [95% CI] | *z* | *p* |  |  |
| Domain type | Intercept (mating domain) | 0.132 | 0.037 | 0.061, 0.204 | 3.610 | <.001 |  |  |
|  | Reproductive domain | 0.004 | 0.075 | -0.143, 0.151 | 0.059 | .953 |  |  |
| Mating domain (MAT), mating behaviors & mating attitudes | | | | | | | | |
| Moderators with too few *k*/*s* (i.e. all potential moderators): | | | | | | | | |
| MAT measure type: behaviors vs attitudes; Sample type: low vs high fertility; Low fertility sample: predominantly students vs non-students; High fertility sample: traditional vs industrialized; Ethnicity: predominantly white vs not; Marriage system: monogamy vs non-monogamy; Publication status: published results; Peer-reviewed; Sexual orientation: heterosexual sample vs gay/mixed/unknown; Normality-transformed variables; Converted effect size; Age controlled for; Non-relevant controls; Sex of experimenter; Illness; Smoker; Condition: baseline vs competition vs courtship. | | | | | | | | |
| Reproductive domain (REP), fertility & reproductive success | | | | | | | | |
| Moderators with too few *k*/*s* (i.e. all potential moderators): | | | | | | | | |
| REP measure type: reproductive success vs fertility; Sample type: low vs high fertility; Low fertility sample: predominantly students vs non-students; High fertility sample: traditional vs industrialized; Ethnicity: predominantly white vs not; Marriage system: monogamy vs non-monogamy; Publication status: published results; Peer-reviewed; Sexual orientation: heterosexual sample vs gay/mixed/unknown; Normality-transformed variables; Converted effect size; Age controlled for; Non-relevant controls; Sex of experimenter; Illness; Smoker; Condition: baseline vs competition vs courtship. | | | | | | | | |
| *Note.* *k* = number of observations, MAT = mating, REP = reproductive, *s* = number of samples. Moderation analyses were only run where each level of the moderator included observations from at least two studies and three independent samples. Analyses were run on the mating measures mating behaviors and mating attitudes, and the reproductive measures fertility and reproductive success when there were enough observations to do so. | | | | | | | | |

Supplementary File 4E

| *Height: moderation analyses. The intercept shows the 'simple effect' for the reference category (specified) and the moderator effect shows the change in effect size for that category relative to the reference category. Moderators are bolded if significant after controlling for multiple comparisons, as indicated by computation of q-values. The full list of q-values can be found in Supplementary File 7.* | | | | | | | | | | | | | | |
| --- | --- | --- | --- | --- | --- | --- | --- | --- | --- | --- | --- | --- | --- | --- |
| Mating vs reproductive domain | | | | | | | | | | | | |  |  |
|  | |  | | B | | SE | [95% CI] | | | *z* | | *p* |  |  |
| Domain type | | Intercept (mating domain) | | 0.049 | | 0.020 | 0.010, 0.088 | | | 2.475 | | .013 |  |  |
|  | | Reproductive domain | | -0.032 | | 0.017 | -0.066, 0.001 | | | -1.900 | | .057 |  |  |
| Mating domain (MAT), mating behaviors & mating attitudes | | | | | | | | | | | | | | |
|  | |  | | B | | SE | | | [95% CI] | *z* | | *p* | | |
| MAT measure type | | Intercept (MAT behaviors) | | 0.054 | | 0.015 | | | 0.024, 0.084 | 3.504 | | .001 | | |
|  | | MAT attitudes | | 0.000 | | 0.021 | | | -0.041, 0.041 | 0.007 | | .995 | | |
| Sample type | | Intercept (low fertility) | | 0.055 | | 0.016 | | | 0.024, 0.086 | 3.456 | | .001 | | |
|  | | High fertility | | 0.031 | | 0.062 | | | -0.092, 0.153 | 0.492 | | .623 | | |
| Sample type: MAT behaviors | | Intercept (low fertility) | | 0.051 | | 0.018 | | | 0.017, 0.086 | 2.923 | | .004 | | |
|  | | High fertility | | 0.034 | | 0.063 | | | -0.090, 0.158 | 0.541 | | .588 | | |
| Low fertility sample | | Intercept (predominantly students) | | 0.065 | | 0.024 | | | 0.018, 0.111 | 2.722 | | .007 | | |
|  | | Non-students/mixed/unknown | | -0.018 | | 0.033 | | | -0.082, 0.047 | -0.537 | | .591 | | |
| Low fertility sample:  MAT attitudes | | Intercept (predominantly students) | | 0.045 | | 0.053 | | | -0.060, 0.150 | 0.843 | | .399 | | |
|  | | Non-students/mixed/unknown | | -0.020 | | 0.058 | | | -0.135, 0.094 | -0.346 | | .729 | | |
| Low fertility sample:  MAT behaviors | | Intercept (predominantly students) | | 0.057 | | 0.026 | | | 0.006, 0.109 | 2.171 | | .030 | | |
|  | | Non-students/mixed/unknown | | -0.010 | | 0.037 | | | -0.082, 0.062 | -0.284 | | .777 | | |
| Ethnicity | | Intercept (predominantly white) | | 0.073 | | 0.023 | | | 0.028, 0.118 | 3.153 | | .002 | | |
|  | | Mixed/other/unknown | | -0.029 | | 0.031 | | | -0.090, 0.032 | -0.918 | | .359 | | |
| Ethnicity: MAT behaviors | | Intercept (predominantly white) | | 0.072 | | 0.026 | | | 0.021, 0.123 | 2.761 | | .006 | | |
|  | | Mixed/other/unknown | | -0.031 | | 0.035 | | | -0.098, 0.037 | -0.890 | | .374 | | |
| Publication status | | Intercept (published results) | | 0.067 | | 0.030 | | | 0.009, 0.126 | 2.269 | | .023 | | |
|  | | Non-published results | | -0.014 | | 0.035 | | | -0.082, 0.054 | -0.403 | | .687 | | |
| Publication status:  MAT behaviors | | Intercept (published results) | | 0.074 | | 0.030 | | | 0.016, 0.133 | 2.486 | | .013 | | |
|  | | Non-published results | | -0.030 | | 0.036 | | | -0.100, 0.040 | -0.833 | | .405 | | |
| Peer-review | | Intercept (peer-reviewed) | | 0.055 | | 0.016 | | | 0.023, 0.087 | 3.348 | | .001 | | |
|  | | Not peer-reviewed | | 0.031 | | 0.058 | | | -0.083, 0.145 | 0.534 | | .593 | | |
| Peer-review: MAT behaviors | | Intercept (peer-reviewed) | | 0.050 | | 0.018 | | | 0.016, 0.085 | 2.867 | | .004 | | |
|  | | Not peer-reviewed | | 0.052 | | 0.066 | | | -0.076, 0.181 | 0.799 | | .424 | | |
| Sexual orientation | | Intercept (heterosexual sample) | | 0.041 | | 0.021 | | | -0.000, 0.082 | 1.954 | | .051 | | |
|  | | Gay/mixed/unknown | | 0.038 | | 0.032 | | | -0.025, 0.101 | 1.180 | | .238 | | |
| Sexual orientation: MAT behaviors | | Intercept (heterosexual sample) | | 0.035 | | 0.023 | | | -0.011, 0.081 | 1.507 | | .132 | | |
|  | | Gay/mixed/unknown | | 0.042 | | 0.035 | | | -0.027, 0.111 | 1.199 | | .231 | | |
| Normality-transformed variables | | Intercept (non-transformed variables) | | 0.049 | | 0.020 | | | 0.011, 0.087 | 2.521 | | .012 | | |
|  | | Transformed variables | | 0.031 | | 0.031 | | | -0.030, 0.091 | 0.995 | | .320 | | |
| Normality-transformed variables: MAT behaviors | | Intercept (non-transformed variables) | | 0.039 | | 0.022 | | | -0.004, 0.081 | 1.796 | | .073 | | |
|  | | Transformed variables | | 0.053 | | 0.036 | | | -0.017, 0.122 | 1.480 | | .139 | | |
| Converted effect size | | Intercept (not converted) | | 0.052 | | 0.017 | | | 0.019, 0.086 | 3.068 | | .002 | | |
|  | | Converted | | 0.048 | | 0.043 | | | -0.036, 0.133 | 1.118 | | .264 | | |
| Converted effect size: MAT behaviors | | Intercept (not converted) | | 0.046 | | 0.019 | | | 0.010, 0.082 | 2.481 | | .013 | | |
|  | | Converted | | 0.055 | | 0.044 | | | -0.031, 0.141 | 1.244 | | .214 | | |
| Age control | | Intercept (age controlled for) | | 0.056 | | 0.018 | | | 0.021, 0.090 | 3.160 | | .002 | | |
|  | | Age not controlled for | | 0.013 | | 0.018 | | | -0.022, 0.048 | 0.708 | | .479 | | |
| Age control: MAT behaviors | | Intercept (age controlled for) | | 0.051 | | 0.017 | | | 0.018, 0.084 | 2.993 | | .003 | | |
|  | | Age not controlled for | | 0.012 | | 0.018 | | | -0.023, 0.047 | 0.663 | | .507 | | |
| Number of measurements | | Intercept (unknown number of measurements) | | 0.047 | | 0.021 | | | 0.006, 0.088 | 2.255 | | .024 | | |
|  | | 2 measurements | | 0.096 | | 0.057 | | | -0.015, 0.207 | 1.702 | | .089 | | |
|  | | 1 measurement: *s* = 1 | |  | |  | | |  |  | |  | | |
| Number of measurements: MAT behaviors | | Intercept (unknown number of measurements) | | 0.051 | | 0.023 | | | 0.006, 0.095 | 2.231 | | .026 | | |
|  | | 2 measurements | | 0.088 | | 0.070 | | | -0.049, 0.226 | 1.264 | | .206 | | |
|  | | 1 measurement: *s* = 1 | |  | |  | | |  |  | |  | | |
| Measurement type | | Intercept (measured) | | 0.057 | | 0.021 | | | 0.016, 0.098 | 2.705 | | .007 | | |
|  | | Self-reported | | 0.001 | | 0.036 | | | -0.069, 0.071 | 0.035 | | .972 | | |
|  | | Unknown measurement type | | 0.010 | | 0.067 | | | -0.122, 0.142 | 0.144 | | .886 | | |
| Measurement type:  MAT behaviors | | Intercept (measured) | | 0.054 | | 0.022 | | | 0.011, 0.097 | 2.455 | | .014 | | |
|  | | Self-reported | | -0.003 | | 0.040 | | | -0.080, 0.075 | -0.073 | | .942 | | |
|  | | Unknown measurement type | | 0.026 | | 0.079 | | | -0.128, 0.180 | 0.329 | | .742 | | |
| Other moderators with too few *k*/*s*: | | | | | | | | | | | | | | |
| High fertility sample: traditional vs industrialized; Marriage system: monogamy vs non-monogamy; Non-relevant controls. | | | | | | | | | | | | | | |
| Reproductive domain (REP), fertility & reproductive success | | | | | | | | | | | | | | |
|  | |  | | B | | SE | | | [95% CI] | *z* | | *p* | | |
| REP measure type | | Intercept (reproductive success) | | -0.031 | | 0.053 | | | -0.135, 0.073 | -0.584 | | .559 | | |
|  | | Fertility | | 0.045 | | 0.054 | | | -0.061, 0.150 | 0.831 | | .406 | | |
| Sample type | | Intercept (low fertility) | | -0.037 | | 0.044 | | | -0.123, 0.050 | -0.825 | | .409 | | |
|  | | High fertility | | 0.071 | | 0.057 | | | -0.041, 0.182 | 1.243 | | .214 | | |
| Sample type: Fertility | | Intercept (low fertility) | | -0.037 | | 0.035 | | | -0.105, 0.031 | -1.060 | | .289 | | |
|  | | High fertility | | 0.090 | | 0.048 | | | -0.004, 0.185 | 1.878 | | .060 | | |
| High fertility sample | | Intercept (traditional) | | 0.029 | | 0.051 | | | -0.071, 0.130 | 0.575 | | .565 | | |
|  | | Industrialized | | 0.011 | | 0.081 | | | -0.147, 0.170 | 0.141 | | .888 | | |
| High fertility sample: Fertility | | Intercept (traditional) | | 0.048 | | 0.042 | | | -0.035, 0.131 | 1.132 | | .258 | | |
|  | | Industrialized | | 0.016 | | 0.057 | | | -0.096, 0.129 | 0.287 | | .774 | | |
| Ethnicity | | Intercept (predominantly white) | | 0.038 | | 0.047 | | | -0.054, 0.130 | 0.808 | | .419 | | |
|  | | Mixed/other/unknown | | -0.049 | | 0.059 | | | -0.165, 0.066 | -0.839 | | .401 | | |
| Ethnicity: Fertility | | Intercept (predominantly white) | | 0.037 | | 0.040 | | | -0.042, 0.116 | 0.924 | | .355 | | |
|  | | Mixed/other/unknown | | -0.044 | | 0.053 | | | -0.147, 0.059 | -0.838 | | .402 | | |
| Marriage system | | Intercept (monogamy) | | -0.010 | | 0.034 | | | -0.078, 0.057 | -0.304 | | .761 | | |
|  | | Non-monogamy | | 0.052 | | 0.060 | | | -0.066, 0.169 | 0.862 | | .389 | | |
| Marriage system: Fertility | | Intercept (monogamy) | | -0.001 | | 0.030 | | | -0.059, 0.057 | -0.034 | | .973 | | |
|  | | Non-monogamy | | 0.052 | | 0.061 | | | -0.067, 0.171 | 0.860 | | .390 | | |
| Publication status | | Intercept (published results) | | -0.023 | | 0.038 | | | -0.098, 0.052 | -0.601 | | .548 | | |
|  | | Non-published results | | 0.064 | | 0.056 | | | -0.046, 0.175 | 1.139 | | .255 | | |
| Publication status: Fertility | | Intercept (published results) | | -0.018 | | 0.036 | | | -0.088, 0.052 | -0.501 | | .617 | | |
|  | | Non-published results | | 0.063 | | 0.052 | | | -0.040, 0.165 | 1.202 | | .229 | | |
| Sexual orientation | | Intercept (heterosexual sample) | | -0.092 | | 0.048 | | | -0.185, 0.001 | -1.939 | | .053 | | |
|  | | **Gay/mixed/unknown** | | **0.135** | | **0.056** | | | **0.026, 0.245** | **2.417** | | **.016** | | |
| Sexual orientation: Fertility | | Intercept (heterosexual sample) | | -0.070 | | 0.041 | | | -0.151, 0.011 | -1.702 | | .089 | | |
|  | | Gay/mixed/unknown | | 0.117 | | 0.050 | | | 0.019, 0.214 | 2.342 | | .019 | | |
| Normality-transformed variables | | Intercept (non-transformed variables) | | -0.005 | | 0.033 | | | -0.069, 0.060 | -0.149 | | .881 | | |
|  | | Transformed variables | | 0.049 | | 0.065 | | | -0.078, 0.175 | 0.756 | | .450 | | |
| Normality-transformed variables: Fertility | | Intercept (non-transformed variables) | | -0.002 | | 0.030 | | | -0.060, 0.056 | -0.081 | | .936 | | |
|  | | Transformed variables | | 0.067 | | 0.064 | | | -0.059, 0.193 | 1.036 | | .300 | | |
| Converted effect size | | Intercept (not converted) | | 0.022 | | 0.034 | | | -0.045, 0.089 | 0.645 | | .519 | | |
|  | | Converted | | -0.048 | | 0.058 | | | -0.161, 0.065 | -0.833 | | .405 | | |
| Converted effect size: Fertility | | Intercept (not converted) | | 0.033 | | 0.032 | | | -0.029, 0.095 | 1.051 | | .293 | | |
|  | | Converted | | -0.072 | | 0.058 | | | -0.186, 0.041 | -1.246 | | .213 | | |
| Converted effect size: Reproductive success | | Intercept (not converted) | | -0.005 | | 0.132 | | | -0.264, 0.254 | -0.037 | | .971 | | |
|  | | Converted | | -0.086 | | 0.187 | | | -0.452, 0.280 | -0.463 | | .644 | | |
| Age control | | Intercept (age controlled for) | | 0.005 | | 0.034 | | | -0.061, 0.071 | 0.154 | | .878 | | |
|  | | Age not controlled for | | -0.032 | | 0.083 | | | -0.195, 0.131 | -0.386 | | .699 | | |
| Non-relevant controls | | Intercept (no non-relevant controls) | | 0.012 | | 0.032 | | | -0.050, 0.075 | 0.387 | | .699 | | |
|  | | Non-relevant controls | | -0.029 | | 0.068 | | | -0.161, 0.103 | -0.428 | | .668 | | |
| Non-relevant controls: Fertility | | Intercept (no non-relevant controls) | | 0.017 | | 0.030 | | | -0.041, 0.076 | 0.585 | | .559 | | |
|  | | Non-relevant controls | | -0.033 | | 0.073 | | | -0.176, 0.111 | -0.443 | | .657 | | |
| Non-relevant controls: Reproductive success | | Intercept (no non-relevant controls) | | 0.017 | | 0.116 | | | -0.210, 0.244 | 0.150 | | .881 | | |
|  | | Non-relevant controls | | -0.166 | | 0.184 | | | -0.526, 0.194 | -0.902 | | .367 | | |
| Measurement type | | Intercept (measured) | | -0.007 | | 0.039 | | | -0.083, 0.070 | -0.167 | | .867 | | |
|  | | Self-reported | | 0.012 | | 0.065 | | | -0.116, 0.140 | 0.178 | | .859 | | |
|  | | Unknown: *s* = 2 | |  | |  | | |  |  | |  | | |
| Measurement type:  Fertility | | Intercept (measured) | | -0.001 | | 0.038 | | | -0.076, 0.073 | -0.037 | | .971 | | |
|  | | Self-reported | | 0.006 | | 0.058 | | | -0.108, 0.121 | 0.105 | | .916 | | |
|  | | Unknown: *s* = 2 | |  | |  | | |  |  | |  | | |
| Other moderators with too few *k*/*s*: | | | | | | | | | | | | | | |
| Low fertility sample: predominantly students vs non-students; Peer-reviewed; Number of measurements. | | | | | | | | | | | | | | |
| *Note.* *k* = number of observations, MAT = mating, REP = reproductive, *s* = number of samples. Moderation analyses were only run where each level of the moderator included observations from at least two studies and three independent samples. Analyses were run on the mating measures mating behaviors and mating attitudes, and the reproductive measures fertility and reproductive success when there were enough observations to do so. | | | | | | | | | | | | | | |

Supplementary File 4F

| *Testosterone levels: moderation analyses. The intercept shows the 'simple effect' for the reference category (specified) and the moderator effect shows the change in effect size for that category relative to the reference category. Moderators are bolded if significant after controlling for multiple comparisons, as indicated by computation of q-values. The full list of q-values can be found in Supplementary File 7.* | | | | | | | | | | | | | | | | | | |
| --- | --- | --- | --- | --- | --- | --- | --- | --- | --- | --- | --- | --- | --- | --- | --- | --- | --- | --- |
| Mating vs reproductive domain | | | | | | | | | | | | | | | |  |  |  |
|  | |  | | B | | SE | | [95% CI] | | | *z* | | | *p* | |  |  |  |
| Domain type | | Intercept (Mating domain) | | 0.093 | | 0.014 | | 0.067, 0.120 | | | 6.893 | | | <.001 | |  |  |  |
|  | | Reproductive domain | | -0.063 | | 0.060 | | -0.181, 0.054 | | | -1.059 | | | .290 | |  |  |  |
| Mating domain (MAT), mating behaviors & mating attitudes | | | | | | | | | | | | | | | | | | |
|  | |  | | B | | SE | | | | [95% CI] | *z* | | | *p* | | | | |
| MAT measure type | | Intercept (MAT behaviors) | | 0.087 | | 0.016 | | 0.056, 0.118 | | | | | 5.460 | | | <.001 |  |  |
|  | | MAT attitudes | | 0.015 | | 0.027 | | -0.038, 0.068 | | | | | 0.569 | | | .569 |  |  |
| Low fertility sample | | Intercept (predominantly students) | | 0.096 | | 0.020 | | 0.057, 0.136 | | | | | 4.755 | | | <.001 |  |  |
|  | | Non-students/mixed/unknown | | 0.012 | | 0.041 | | -0.069, 0.094 | | | | | 0.298 | | | .766 |  |  |
| Low fertility sample:  MAT behaviors | | Intercept (predominantly students) | | 0.080 | | 0.024 | | 0.032, 0.128 | | | | | 3.288 | | | .001 |  |  |
|  | | Non-students/mixed/unknown | | 0.024 | | 0.039 | | -0.053, 0.101 | | | | | 0.610 | | | .542 |  |  |
| Ethnicity | | Intercept (predominantly white) | | 0.104 | | 0.023 | | 0.058, 0.150 | | | | | 4.436 | | | <.001 |  |  |
|  | | Mixed/other/unknown | | -0.016 | | 0.030 | | -0.074, 0.043 | | | | | -0.530 | | | .596 |  |  |
| Ethnicity: MAT attitudes | | Intercept (predominantly white) | | 0.126 | | 0.060 | | 0.008, 0.245 | | | | | 2.090 | | | .037 |  |  |
|  | | Mixed/other/unknown | | -0.045 | | 0.074 | | -0.189, 0.099 | | | | | -0.614 | | | .540 |  |  |
| Ethnicity: MAT behaviors | | Intercept (predominantly white) | | 0.099 | | 0.022 | | 0.056, 0.142 | | | | | 4.497 | | | <.001 |  |  |
|  | | Mixed/other/unknown | | -0.024 | | 0.025 | | -0.074, 0.026 | | | | | -0.943 | | | .346 |  |  |
| Publication status | | Intercept (published results) | | 0.097 | | 0.017 | | 0.064, 0.130 | | | | | 5.736 | | | <.001 |  |  |
|  | | Non-published results | | 0.012 | | 0.039 | | -0.088, 0.065 | | | | | 0.303 | | | .762 |  |  |
| Publication status:  MAT behaviors | | Intercept (published results) | | 0.082 | | 0.014 | | 0.054, 0.110 | | | | | 5.703 | | | <.001 |  |  |
|  | | Non-published results | | 0.019 | | 0.041 | | -0.061, 0.098 | | | | | 0.465 | | | .642 |  |  |
| Sexual orientation | | Intercept (heterosexual sample) | | 0.125 | | 0.016 | | 0.092, 0.157 | | | | | 7.578 | | | <.001 |  |  |
|  | | **Gay/mixed/unknown** | | **-0.059** | | **0.020** | | **-0.098, -0.021** | | | | | **-2.994** | | | **.003** |  |  |
| Sexual orientation: MAT attitudes | | Intercept (heterosexual sample) | | 0.165 | | 0.059 | | 0.050, 0.281 | | | | | 2.803 | | | .005 |  |  |
|  | | Gay/mixed/unknown | | -0.108 | | 0.076 | | -0.256, 0.041 | | | | | -1.419 | | | .156 |  |  |
| Sexual orientation: MAT behaviors | | Intercept (heterosexual sample) | | 0.110 | | 0.020 | | 0.071, 0.149 | | | | | 5.529 | | | <.001 |  |  |
|  | | Gay/mixed/unknown | | -0.042 | | 0.024 | | -0.089, 0.005 | | | | | -1.751 | | | .080 |  |  |
| Normality-transformed variables | | Intercept (non-transformed variables) | | 0.073 | | 0.010 | | 0.053, 0.093 | | | | | 7.172 | | | <.001 |  |  |
|  | | **Transformed variables** | | **0.057** | | **0.023** | | **0.011, 0.103** | | | | | **2.445** | | | **.015** |  |  |
| Normality-transformed variables: MAT behaviors | | Intercept (non-transformed variables) | | 0.074 | | 0.012 | | 0.050, 0.098 | | | | | 5.995 | | | <.001 |  |  |
|  | | Transformed variables | | 0.036 | | 0.028 | | -0.018, 0.091 | | | | | 1.306 | | | .192 |  |  |
| Converted effect size | | Intercept (not converted) | | 0.091 | | 0.016 | | 0.061, 0.121 | | | | | 5.863 | | | <.001 |  |  |
|  | | Converted | | 0.029 | | 0.043 | | -0.056, 0.114 | | | | | 0.665 | | | .506 |  |  |
| Converted effect size: MAT behaviors | | Intercept (not converted) | | 0.078 | | 0.013 | | 0.054, 0.103 | | | | | 6.227 | | | <.001 |  |  |
|  | | Converted | | 0.058 | | 0.046 | | -0.032, 0.147 | | | | | 1.266 | | | .206 |  |  |
| Age control | | Intercept (age controlled for) | | 0.098 | | 0.022 | | 0.054, 0.142 | | | | | 4.393 | | | <.001 |  |  |
|  | | Age not controlled for | | -0.011 | | 0.032 | | -0.074, 0.052 | | | | | -0.346 | | | .730 |  |  |
| Age control: MAT behaviors | | Intercept (age controlled for) | | 0.107 | | 0.029 | | 0.050, 0.163 | | | | | 3.690 | | | <.001 |  |  |
|  | | Age not controlled for | | -0.029 | | 0.041 | | -0.109, 0.051 | | | | | -0.708 | | | .479 |  |  |
| Non-relevant controls | | Intercept (no non-relevant controls) | | 0.088 | | 0.015 | | 0.059, 0.117 | | | | | 5.954 | | | <.001 |  |  |
|  | | Non-relevant controls | | 0.042 | | 0.041 | | -0.039, 0.122 | | | | | 1.012 | | | .312 |  |  |
| Time of day | | Intercept (AM) | | 0.090 | | 0.023 | | 0.045, 0.135 | | | | | 3.899 | | | <.001 |  |  |
|  | | PM | | 0.016 | | 0.028 | | -0.038, 0.070 | | | | | 0.568 | | | .570 |  |  |
|  | | Mixed/unknown | | -0.014 | | 0.046 | | -0.105, 0.076 | | | | | -0.309 | | | .757 |  |  |
| Time of day: MAT attitudes | | Intercept (AM) | | 0.069 | | 0.045 | | -0.019, 0.157 | | | | | 1.543 | | | .123 |  |  |
|  | | PM | | 0.038 | | 0.047 | | -0.055, 0.130 | | | | | 0.794 | | | .427 |  |  |
|  | | Mixed/unknown: *s* = 2 | |  | |  | |  | | | | |  | | |  |  |  |
| Time of day: MAT behaviors | | Intercept (AM) | | 0.088 | | 0.024 | | 0.041, 0.134 | | | | | 3.672 | | | <.001 |  |  |
|  | | PM | | 0.003 | | 0.036 | | -0.068, 0.074 | | | | | 0.087 | | | .931 |  |  |
|  | | Mixed/unknown | | -0.017 | | 0.049 | | -0.114, 0.079 | | | | | -0.350 | | | .726 |  |  |
| Blood contamination | | Intercept (checked) | | 0.142 | | 0.031 | | 0.080, 0.203 | | | | | 4.533 | | | <.001 |  |  |
|  | | Not checked/unknown | | -0.061 | | 0.036 | | -0.133, 0.010 | | | | | -1.694 | | | .090 |  |  |
| Blood contamination: MAT behaviors | | Intercept (checked) | | 0.130 | | 0.032 | | 0.067, 0.193 | | | | | 4.036 | | | <.001 |  |  |
|  | | Not checked/unknown | | -0.064 | | 0.040 | | -0.141, 0.014 | | | | | -1.599 | | | .110 |  |  |
| Fatherhood status | | Intercept (non-fathers) | | 0.069 | | 0.025 | | 0.021, 0.117 | | | | | 2.831 | | | .005 |  |  |
|  | | Mixed/unknown | | 0.040 | | 0.031 | | -0.021, 0.101 | | | | | 1.286 | | | .198 |  |  |
|  | | Fathers: *s* = 0 | |  | |  | |  | | | | |  | | |  |  |  |
| Fatherhood status: MAT attitudes | | Intercept (non-fathers) | | 0.072 | | 0.054 | | -0.033, 0.177 | | | | | 1.339 | | | .181 |  |  |
|  | | Mixed/unknown | | 0.061 | | 0.081 | | -0.097, 0.219 | | | | | 0.755 | | | .450 |  |  |
|  | | Fathers: *s* = 0 | |  | |  | |  | | | | |  | | |  |  |  |
| Fatherhood status: MAT behaviors | | Intercept (non-fathers) | | 0.055 | | 0.031 | | -0.005, 0.115 | | | | | 1.792 | | | .073 |  |  |
|  | | Mixed/unknown | | 0.043 | | 0.036 | | -0.027, 0.114 | | | | | 1.203 | | | .229 |  |  |
|  | | Fathers: *s* = 0 | |  | |  | |  | | | | |  | | |  |  |  |
| Other moderators with too few *k*/*s*: | | | | | | | | | | | | | | | | | | |
| Sample type: low vs high fertility; High fertility sample: traditional vs industrialized; Marriage system: monogamy vs non-monogamy; Peer-reviewed; How assayed: blood vs saliva; Relationship status | | | | | | | | | | | | | | | | | | |
| Reproductive domain (REP), fertility & reproductive success | | | | | | | | | | | | | | | | | |  |
| Moderators with too few *k*/*s* (i.e. all potential moderators): | | | | | | | | | | | | | | | | | |  |
| REP measure type (reproductive success vs fertility; Sample type: low vs high fertility; Low fertility sample: predominantly students vs non-students; High fertility sample: traditional vs industrialized; Ethnicity: predominantly white vs not; Marriage system: monogamy vs non-monogamy; Publication status: published results; Peer-reviewed; Sexual orientation: heterosexual sample vs gay/mixed/unknown; Normality-transformed variables; Converted effect size; Age controlled for; Non-relevant controls; How assayed: blood vs saliva; Time of day: AM vs PM vs mixed/unknown; Fatherhood status; Relationship status | | | | | | | | | | | | | | | | | |  |
| *Note.* *k* = number of observations, MAT = mating, REP = reproductive, *s* = number of samples. Moderation analyses were only run where each level of the moderator included observations from at least two studies and three independent samples. Analyses were run on the mating measures mating behaviors and mating attitudes, and the reproductive measures fertility and reproductive success when there were enough observations to do so. | | | | | | | | | | | | | | | | | |  |
