# Supplementary material for: A meta-analysis of the association between male dimorphism and fitness outcomes in humans
Source: eLife. 2022 Feb 18;11:e65031. doi: 10.7554/eLife.65031 (PMC9106334; doi:10.7554/eLife.65031)
Supplement: Supplementary file 5. [file elife-65031-supp5.docx]

Supplementary File 5A

|  | | | |
| --- | --- | --- | --- |
| *Mating domain, reproductive domain, and offspring mortality domain predicted by global masculinity. Pearson’s r (95% CI); p value for meta-analytic effect, q-value (correcting for multiple comparisons); number of observations (k), samples (s), and unique participants (n); test for heterogeneity (Q), p value for heterogeneity. Statistically significant meta-analytic associations are bolded if still significant after controlling for multiple comparisons.* | | | |
|  | Mating domain | Reproductive domain | Offspring mortality domain |
| Sample |  |  |  |
| All samples | ***r* = .090 (0.071, 0.110), *p* < .001, *q* = .001** *k* = 371, *s* = 70, *n* = 117481 Q(df = 370) = 1108.213, *p* < .001 | *r* = .047 (0.004, 0.090),  *p* = .033, *q* = .080 *k* = 81, *s* = 36, *n* = 107848 Q(df = 80) = 628.883, *p* < .001 | *r* = .002 (-0.011, 0.015), *p* = .782, *q* = .475 *k* = 22, *s* = 13, *n* = 21991 Q(df = 21) = 14.765, *p* = .835 |
| *Note.* k = number of observations; n = number of unique participants; Q = Cochran’s Q test of heterogeneity; s = number of samples. | | | |

Supplementary File 5B

|  | | | | | | | | |
| --- | --- | --- | --- | --- | --- | --- | --- | --- |
| *Global masculinity: moderation analyses. The intercept shows the 'simple effect' for the reference category (specified) and the moderator effect shows the change in effect size for that category relative to the reference category. Moderators are bolded if significant after controlling for multiple comparisons, as indicated by computation of q-values. The full list of q-values can be found in Supplementary File 7.* | | | | | | | | |
| Global masculinity | | | | | | |  |  |
|  |  | B | SE | [95% CI] | *z* | *p* |  |  |
| Domain type | **Intercept (mating domain)** | **0.083** | **0.010** | **0.062, 0.103** | **8.022** | **<.001** |  |  |
|  | Reproductive domain | 0.004 | 0.003 | -0.003, 0.011 | 1.209 | .227 |  |  |
|  | Offspring mortality domain | -0.054 | 0.030 | -0.113, 0.005 | -1.794 | .073 |  |  |
| Mating domain: moderation analyses of type of masculinity | | | | | | | | |
|  |  | B | SE | [95% CI] | *z* | *p* |  |  |
| Masculinity type | **Intercept (body masculinity)** | **0.108** | **0.012** | **0.085, 0.132** | **8.951** | **<.001** |  |  |
|  | **2D:4D** | **-0.060** | **0.018** | **-0.096, -0.025** | **-3.325** | **.001** |  |  |
|  | **Facial masculinity** | **-0.054** | **0.023** | **-0.099, -0.009** | **-2.354** | **.019** |  |  |
|  | **Height** | **-0.020** | **0.009** | **-0.037, -0.002** | **-2.235** | **.025** |  |  |
|  | Testosterone levels | -0.009 | 0.020 | -0.047, 0.030 | -0.452 | .652 |  |  |
|  | Voice pitch | 0.033 | 0.041 | -0.047, 0.113 | 0.814 | .416 |  |  |
| Reproductive domain: moderation analyses of type of masculinity | | | | | | | | |
|  |  | B | SE | [95% CI] | *z* | *p* |  |  |
| Masculinity type | **Intercept (body masculinity)** | **0.117** | **0.039** | **0.040, 0.194** | **2.980** | **.003** |  |  |
|  | 2D:4D | -0.042 | 0.052 | -0.143, 0.059 | -0.812 | .417 |  |  |
|  | Facial masculinity | -0.030 | 0.071 | -0.170, 0.110 | -0.417 | .677 |  |  |
|  | **Height** | **-0.107** | **0.038** | **-0.181, -0.033** | **-2.817** | **.005** |  |  |
|  | Testosterone levels | -0.028 | 0.069 | -0.163, 0.107 | -0.403 | .687 |  |  |
|  | Voice pitch | -0.032 | 0.079 | -0.187, 0.122 | -0.411 | .681 |  |  |
| *Note.* Moderation analyses were only run where each level of the moderator included observations from at least two studies and three independent samples. | | | | | | | | |
