# Supplementary material for: A meta-analysis of the association between male dimorphism and fitness outcomes in humans
Source: eLife. 2022 Feb 18;11:e65031. doi: 10.7554/eLife.65031 (PMC9106334; doi:10.7554/eLife.65031)
Supplement: Supplementary file 6. [file elife-65031-supp6.docx]

Supplementary File 6A

*
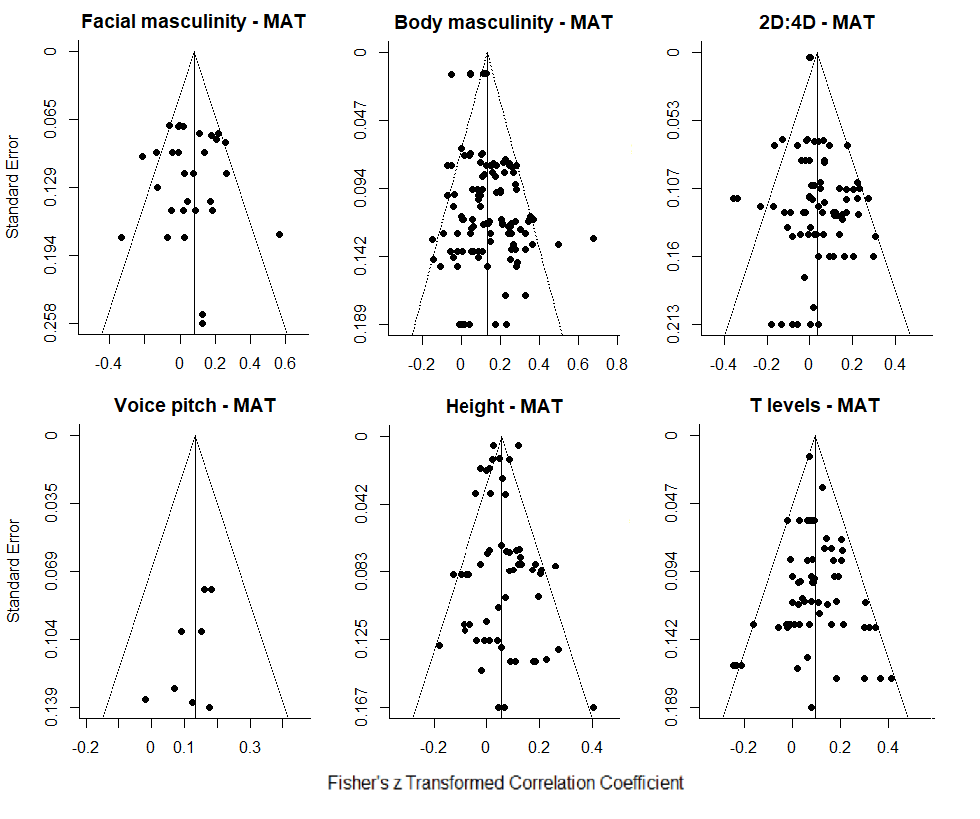
*

Funnel plots of effect sizes for mating measures (MAT). T = testosterone.

Supplementary File 6B


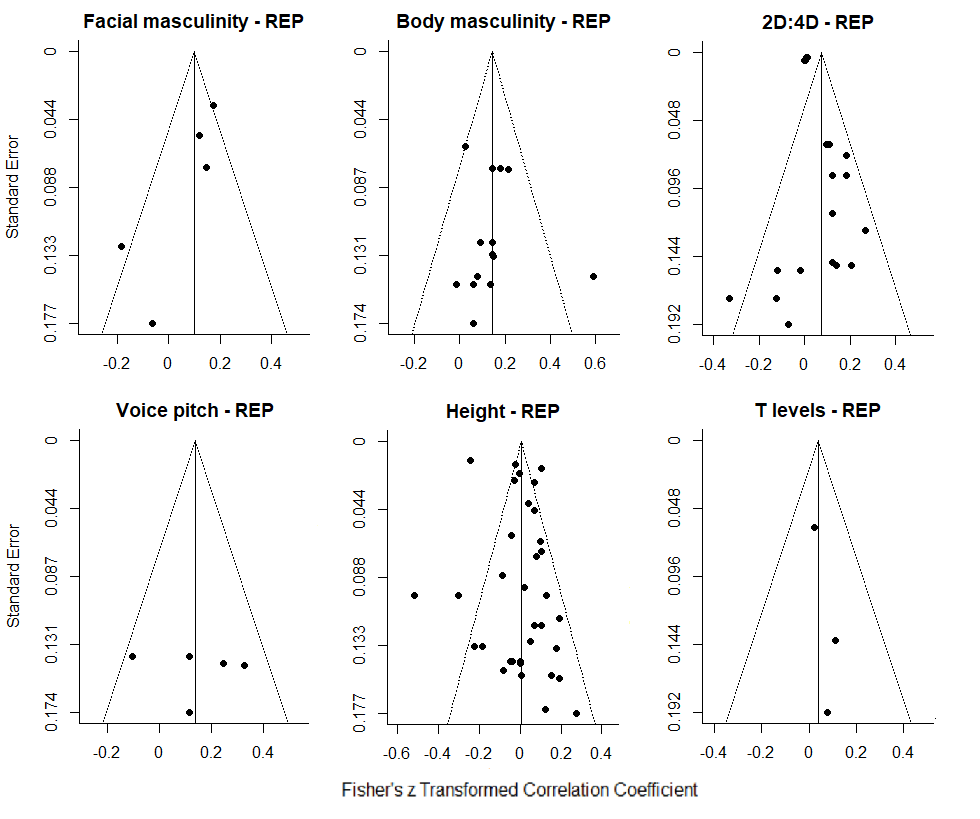


Funnel plots of effect sizes for reproductive measures (REP). T = testosterone levels.
