# Supplementary material for: A meta-analysis of the association between male dimorphism and fitness outcomes in humans
Source: eLife. 2022 Feb 18;11:e65031. doi: 10.7554/eLife.65031 (PMC9106334; doi:10.7554/eLife.65031)
Supplement: Supplementary file 7. [file elife-65031-supp7.docx]

Supplementary File 7

*Output for q-value computation for all analyses*
